# Epicardial adipose tissue and subclinical incident atrial fibrillation as detected by continuous monitoring: a cardiac magnetic resonance imaging study

**DOI:** 10.1007/s10554-023-03029-z

**Published:** 2024-01-21

**Authors:** Eva Guldberg, Søren Zöga Diederichsen, Ketil Jørgen Haugan, Axel Brandes, Claus Graff, Derk Krieger, Morten Salling Olesen, Søren Højberg, Lars Køber, Niels Vejlstrup, Litten Bertelsen, Jesper Hastrup Svendsen

**Affiliations:** 1 Department of Cardiology, Copenhagen University Hospital – Rigshospitalet, Inge Lehmanns Vej 7, 2100 Copenhagen, Denmark; 2 Department of Cardiology, Zealand University Hospital - Roskilde, Roskilde, Denmark; 3 Department of Cardiology, Odense University Hospital, Odense, Denmark; 4 Faculty of Health Sciences, Department of Clinical Research, University of Southern Denmark, Odense, Denmark; 5 Department of Cardiology, Esbjerg Hospital – University Hospital of Southern Denmark, Esbjerg, Denmark; 6 Department of Health Science and Technology, Aalborg University, Aalborg, Denmark; 7 Mohammed Bin Rashid University, Mediclinic Parkview Hospital, Dubai, UAE; 8 Laboratory for Molecular Cardiology, Department of Cardiology, Copenhagen University Hospital – Rigshospitalet, Copenhagen, Denmark; 9 Faculty of Health and Medical Sciences, Department of Biomedical Sciences, University of Copenhagen, Copenhagen, Denmark; 10 Department of Cardiology, Copenhagen University Hospital - Bispebjerg and Frederiksberg, Copenhagen, Denmark; 11 Faculty of Health and Medical Sciences, Department of Clinical Medicine, University of Copenhagen, Copenhagen, Denmark

**Keywords:** Epicardial adipose tissue, Atrial fibrillation, Cardiac magnetic resonance

## Abstract

Epicardial adipose tissue (EAT) has endocrine and paracrine functions and has been associated with metabolic and cardiovascular disease. This study aimed to investigate the association between EAT, determined by cardiac magnetic resonance imaging (CMR), and incident atrial fibrillation (AF) following long-term continuous heart rhythm monitoring by implantable loop recorder (ILR). This study is a sub-study of the LOOP study. In total, 203 participants without a history of AF received an ILR and underwent advanced CMR. All participants were at least 70 years of age at inclusion and had at least one of the following conditions: hypertension, diabetes, previous stroke, or heart failure. Volumetric measurements of atrial- and ventricular EAT were derived from CMR and the time to incident AF was subsequently determined. A total of 78 participants (38%) were diagnosed with subclinical AF during a median of 40 (37–42) months of continuous monitoring. In multivariable Cox regression analyses adjusted for age, sex, and various comorbidities, we found EAT indexed to body surface area to be independently associated with the time to AF with hazard ratios (95% confidence intervals) up to 2.93 (1.36–6.34); p = 0.01 when analyzing the risk of new-onset AF episodes lasting ≥ 24 h. Atrial EAT assessed by volumetric measurements on CMR images was significantly associated with the incident AF episodes as detected by ILR.

**Supplementary Information:**

The online version contains supplementary material available at 10.1007/s10554-023-03029-z.

## Introduction

Atrial fibrillation (AF) is the most common cardiac arrhythmia worldwide and is associated with a substantially increased risk of morbidity and mortality, including heart failure and stroke [1–3]. The risk of AF increases with age, and the risk of asymptomatic episodes further increases in the elderly [4], whereas adverse outcomes can arise independently of symptoms [3].

The heart is surrounded by two layers of pericardium which comprises the pericardial space containing a fluid. The adipose tissue in proximity to the myocardium is anatomically defined as epicardial adipose tissue (EAT). The adipose tissue external to the parietal pericardium within the mediastinal cavity is defined as paracardial adipose tissue, which combined with EAT constitutes pericardial adipose tissue. The association between AF and EAT has long been of great interest due to the heterogeneous composition and functions of EAT.

Previous studies have mainly focused on either paracardial adipose tissue or pericardial adipose tissue. Recent studies suggest that EAT has the strongest association with multiple conditions such as insulin resistance, diabetes, hypertension, dyslipidemia, AF, and increased risk of both cardiovascular disease and stroke [1, 3, 5].

There are multiple physiological functions of EAT in relation to AF, and there seems to be a dichotomy in the physiological effects of EAT [6, 7]. It is believed to provide both thermoregulation and mechanical protection to the heart and coronary arteries in healthy human beings [8, 9], but the role of EAT in the development and severity of AF has been investigated and suggests deleterious effects of EAT.

Since EAT shares blood circulation with the myocardium [8], it offers the opportunity for paracrine- and endocrine signaling [7]. The paracrine effect of secretion of the hormone resistin has been demonstrated to cause contractile dysfunction due to abnormal calcium regulation [7, 10]. EAT is also thought to inhibit the cardiac electrical conduction through the formation of fibrosis [7, 11] and the pro-inflammatory components of EAT have furthermore been demonstrated to play a role in the association with AF through the secretion of adipocytokines [7, 8, 12]. Multiple studies have shown EAT to increase cholinergic activity [7, 12]. This is believed to be mediated by ganglionated plexi embedded in the EAT and an expansion of EAT can lead to a dysregulation of the autonomic nervous system, again contributing to a pro-inflammatory state [7, 13].

While EAT assessed by cardiac magnetic resonance imaging (CMR) has been found predictive of clinical AF [6, 7], the association with subclinical AF has not yet been established. Furthermore, the interplay between EAT and atrial remodeling has not been fully elucidated.

This study aimed to quantify the volume of EAT by CMR imaging in an at-risk population and analyze its significance when several different clinical parameters were taken into consideration. We wanted to divide the EAT into atrial- and ventricular EAT and investigate the correlation between EAT and incident subclinical AF as registered by implantable loop recorders.

## Methods

This study is based on the LOOP study (NCT02036450), where participants from the general population were randomly selected and invited to participate. A detailed description of the study design has previously been published [14]. In short, all participants were at least 70 years old at inclusion and had at least one condition associated with an increased risk of stroke, i.e., hypertension, diabetes, previous stroke, or heart failure. Exclusion criteria were among others known history of AF, ongoing oral anticoagulation therapy, or contraindication to oral anticoagulation. Included participants were randomized to receive standard care versus long-term monitoring using an implantable loop recorder (ILR), which is considered the gold standard for identifying subclinical AF. All new-onset ILR-detected AF episodes lasting ≥ 6 min during the LOOP study were independently adjudicated by at least two consultant cardiologists. Agreement between the two cardiologists was required. In case of disagreement, a third cardiologist was involved to achieve a final judgment.

The current study is a sub-study performed on participants with available CMR scans from inclusion in the LOOP study. Participants who were randomized to ILR in the LOOP study were invited to also participate in the CMR sub-study. Details of the CMR scans have been published previously [15, 16]. In short, eligible participants were scanned within a few weeks of inclusion. The exclusion criteria for the CMR study were contraindications to magnetic resonance imaging.

The LOOP study has been approved by the Ethics Committee for the Capital Region of Denmark (protocol number H-4-2013-025). All study participants provided oral and written informed consent.

### Image acquisition

All scans were performed on a 1.5-T scanner (Espree, Siemens Healthcare, Erlangen, Germany) [16]. The scan included long-axis images and an axial cine stack covering the base of the heart to the aortic arch. All participants exhibited normal sinus rhythm during CMR acquisition.

Details of scanning parameters are provided in *Supplementary material.*

### Image analyses

**Fig. 1:**
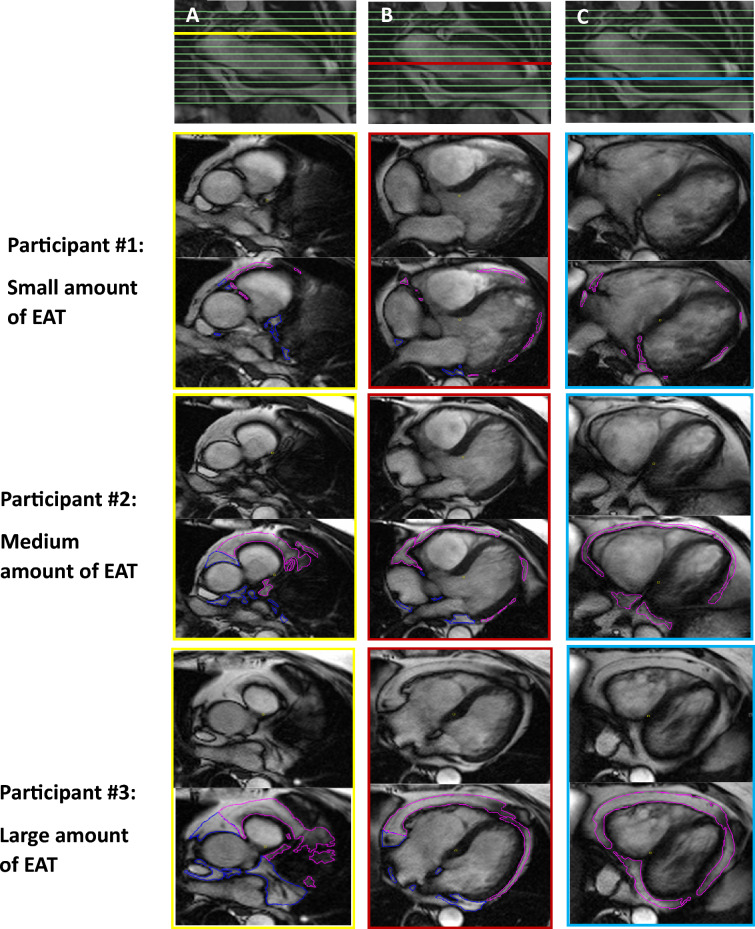
Analyses of cardiac magnetic resonance imaging. Image **A**–**C**: To ensure sufficient dimensional orientation, a two-chamber image is used as reference. In this figure, the reference is presented in three different slices marked with yellow, red, and turquoise. Below these images, the axial cine images before and after contouring of the epicardial adipose tissue (EAT) are presented. The volumetric measurements are performed on all slices in a full image stack for all participants, where it is divided into ventricular EAT (pink) and atrial (blue)

All volumetric analyses were performed in the software CVI (v. 5.6.5, Circle Cardiovascular Imaging Inc., Calgary, Canada). EAT was manually depicted using the pericardium as the outer border. The pericardium itself was not included and the separation of EAT from epicardial fluid was visually assessed. Consecutive volumes of atrial- and ventricular EAT were measured separately on axial cine images in a cranial—caudal direction. The most cranial image was defined as the image just below the separation of the pulmonary arteries. The most caudal image was defined as the image just below the ventricles which, when visually assessed, still followed the cardiac cycle. All volumetric measurements of both atrial- and ventricular EAT were performed during the ventricular end-diastolic phase. The mitral- and tricuspid annular rings defined the borders between the atria and ventricle and separated EAT into atrial and ventricular EAT, which is illustrated in Fig. 1. To distinguish EAT from paracardial adipose tissue, the movements of the adipose tissue were assessed following the contraction and dilation during the heart cycle. To illustrate these movements of adipose tissue, the cardiac cycle on one image slice is visualized in Video S2 in *Supplementary material*. Long-axis images were used as cross-reference to ensure sufficient dimensional understanding, and hereby support a correct separation of atrial EAT from ventricular EAT.

CMR analyses of EAT in this study were performed by the same investigator (EG), who was blinded to clinical data including the presence of AF. All other results were derived from previous analyses [16]. Left atrial volumes were derived from the axial stack. Left atrial total emptying fraction (LATEF) was defined as (left atrial maximal volume–left atrial minimum volume)/left atrial maximal volume), i.e., (LAMAX-LAMIN)/LAMAX. In this study, we included LAMIN rather than LAMAX, as a parameter of left atrial volume since a previous CMR sub-study of the LOOP study found LAMIN to be a more reliable marker for subclinical AF [16].

To ensure reproducibility, interobserver and intraobserver variability were assessed in 20 randomly selected participants. For this assessment, the intraclass correlation coefficient (ICC) was evaluated and good correlation was defined as ICC > 0.75.

### AF burden

AF burden was defined as the cumulative duration of all AF episodes from the first adjudicated episode until the end of monitoring divided by the cumulative duration of monitoring.

## Statistics

Variables are expressed as mean and standard deviation if they were visually assessed as normally distributed. If non-normally distributed, variables are expressed as median and interquartile range. Student’s *t*-test was used to compare normally distributed groups. Wilcoxon rank test was used for non-normally distributed groups.

Correlations between variables were assessed visually with a series of scatterplots.

We performed Cox regression analyses as stepwise constructions with time to AF debut (AF episode ≥ 6 min), first AF episode ≥ 5.5 h, and first episode ≥ 24 h, respectively. Initially, the EAT surrounding the atria and ventricle was analyzed in univariable Cox regressions separately.

Subsequently, multivariable Cox regression analyses were adjusted for the following variables: *Model 1* was adjusted for the baseline characteristics age and sex as well as comorbidities from the CHA_2_DS_2_-VASc-score (heart failure, hypertension, diabetes, previous stroke, cardiac valvular disease, and previous acute myocardial infarction and/or coronary artery bypass graft surgery). *Model 2* included model 1 as well as LAMIN (Table S6). *Model 3* included model 1 as well as LATEF as predictor variables (Table S7). Separate models were constructed for LATEF and LAMIN to avoid collinearity.

Both EAT and LAMIN were indexed to body surface area in all analyses as indicated by i in the supplementary tables. These volumes were indexed to ensure that the participant’s body composition did not influence on the results.

We also evaluated the possible added discriminative value of atrial- and ventricular EAT over the already established prediction score for clinical AF, CHARGE AF [17]. This analysis was conducted based on baseline assessments, though race was not included since all participants were of European ancestry. We used cause-specific Cox regression models to analyze the risk of getting AF episodes lasting ≥ 6 min, ≥ 5.5 h, and ≥ 24 h. The area under the receiver operating characteristics curve was calculated with the reference based on CHARGE AF.

A two-sided p-value of ≤ 0.05 was considered statistically significant. All analyses were performed in the software RStudio 2022.02.2.

## Results

In total, 203 participants were included in the study. A total of 78 participants (38%) had adjudicated AF episodes during a median of 40 (37–42) months of continuous monitoring. All AF episodes were detected by an ILR and are hereby considered as subclinical AF. AF episodes were divided into 3 groups based on AF durations. All 78 patients had AF for at least 6 min (definition of AF in the LOOP study [14]). Of these, 40 patients (20%) had AF lasting 5.5 h or longer. Lastly, 15 (7%) of these participants had AF episodes of 24 h or more.

The baseline characteristics for all participants are summarized in Table 1*.*

**Table 1:** Baseline characteristics

Variable	Total
Male, n (%)	129 (63.2)
Age (years), mean (SD)	76.2 (4.2)
Medical history	
Hypertension, n (%)	185 (90.7)
Diabetes mellitus, n (%)	64 (31.4)
Congestive heart failure, n (%)	7 (3.4)
Previous stroke, n (%)	39 (19.1)
Previous transient ischemic attack, n (%)	23 (11.3)
Previous acute myocardial infarction, n (%)	19 (9.3)
Previous CABG, n (%)	9 (4.4)
Valvular disease, n (%)	8 (3.9)
COPD, n (%)	12 (5.9)
CHA2DS2-VASc-score, mean (SD)	2.9 (1.2)
Medical treatment	
Beta blockers n (%)	41 (20.1)
Calcium blockers n (%)	78 (38.2)
Renin-angiotensin-system medication n (%)	120 (58.8)
Statins, n (%)	114 (55.9)
Diuretics, n (%)	65 (31.9)
Platelet inhibitors, n (%)	107 (52.5)
Antidiabetics, n (%)	57 (27.9)
Physical examination	
Systolic blood pressure (mmHg), mean (SD)	148.3 (17.8)
Diastolic blood pressure (mmHg), mean (SD)	84.1 (10.9)
Pulse rate (bpm), mean (SD)	70.9 (11.9)
Height (cm), mean (SD)	172.1 (9)
Weight (kg), mean (SD)	83.5 (15.6)
BMI (kg/cm^2^), mean (SD)	28.1 (4.7)

This table presents the baseline characteristics of all 203 participants

*CABG* coronary artery bypass grafting surgery, *COPD* chronic obstructive pulmonary disease, *CHA2DS2-VASc-score* point-base system to stratify the risk of stroke, *BMI* body mass index

The baseline characteristics are furthermore illustrated in *Supplementary Material* where *Table S2* and *s3* are divided into groups of small- and large amounts of atrial- and ventricular EAT, respectively.

The CMR measurements used to calculate the ICC are summarized in Supplementary material (Table S1). Interobserver ICC for measurement of ventricular EAT was 0.91 (95% confidence interval: 0.78–0.96) and intraobserver ICC for ventricular EAT was 0.96 (0.91–0.99). Interobserver ICC for measurement of atrial EAT was 0.85 (0.66–0.94) and intraobserver ICC for atrial EAT was 0.88 (0.73–0.95). As mentioned, the ICC for atrial measurements is generally lower than for the ventricular measurements. Since there is a smaller amount of atrial EAT, even minor differences during the measuring process have a higher impact on the ICC. Furthermore, atrial EAT is more difficult to measure, since the boundaries on the CMR are harder to distinguish. An overview of the participants CMR characteristics can be found in Table 2*.*

**Table 2:** CMR characteristics

	Total
Atrial EATi (ml), median [IQR]	11 [7.5, 16.7]
Ventricular EATi (ml), median [IQR]	31.6 [22.9, 44.1]
LAMINi (ml/m2), median [IQR]	24.9 [19.7, 30.7]
LAMAXi (ml/m2), mean (SD)	50.4 (12.9)
LV end diastolic volume (ml/m2), mean (SD)	73.3 (15.6)
LV end systolic volume (ml/m2), median [IQR]	23.4 [18.8, 30.4]
LA total emptying fraction (%), mean (SD)	48.5 (7)
LV total emptying fraction (%), mean (SD)	66 (7.4)

This table presents the cardiac magnetic resonance (CMR) characteristics of all 203 participants

*EATi* epicardial adipose tissue indexed to body surface area, *LAMINi* left atrial minimum volume indexed to body surface area, *LAMAXi* left atrial maximal volume indexed to body surface area, *LV* left ventricle, *LA* left atrium

### Association between EAT and AF episode duration

In univariable Cox regressions, we did not find a significant association between EAT and any of the episode durations (Table S4).

After adjustment for baseline characteristics in model 1, both atrial EAT and ventricular EAT were significantly associated with time to AF episodes ≥ 5.5 h and ≥ 24 h. The strongest associations were seen for atrial EAT with the (hazard ratio) HR of 1.63 [confidence interval (CI) (1.0–2.64); p = 0.05] for ≥ 5.5 h and the HR of 2.23 [CI (1.1–4.51); p = 0.03] for AF episodes ≥ 24 h. Similar signals were observed for ventricular EAT with HR of 1.09 [CI (1.0–1.41); p = 0.05] and 1.29 [CI (1.0–1.65); p = 0.05] for ≥ 5.5 h and ≥ 24 h, respectively (Table S5).

**Fig. 2:**
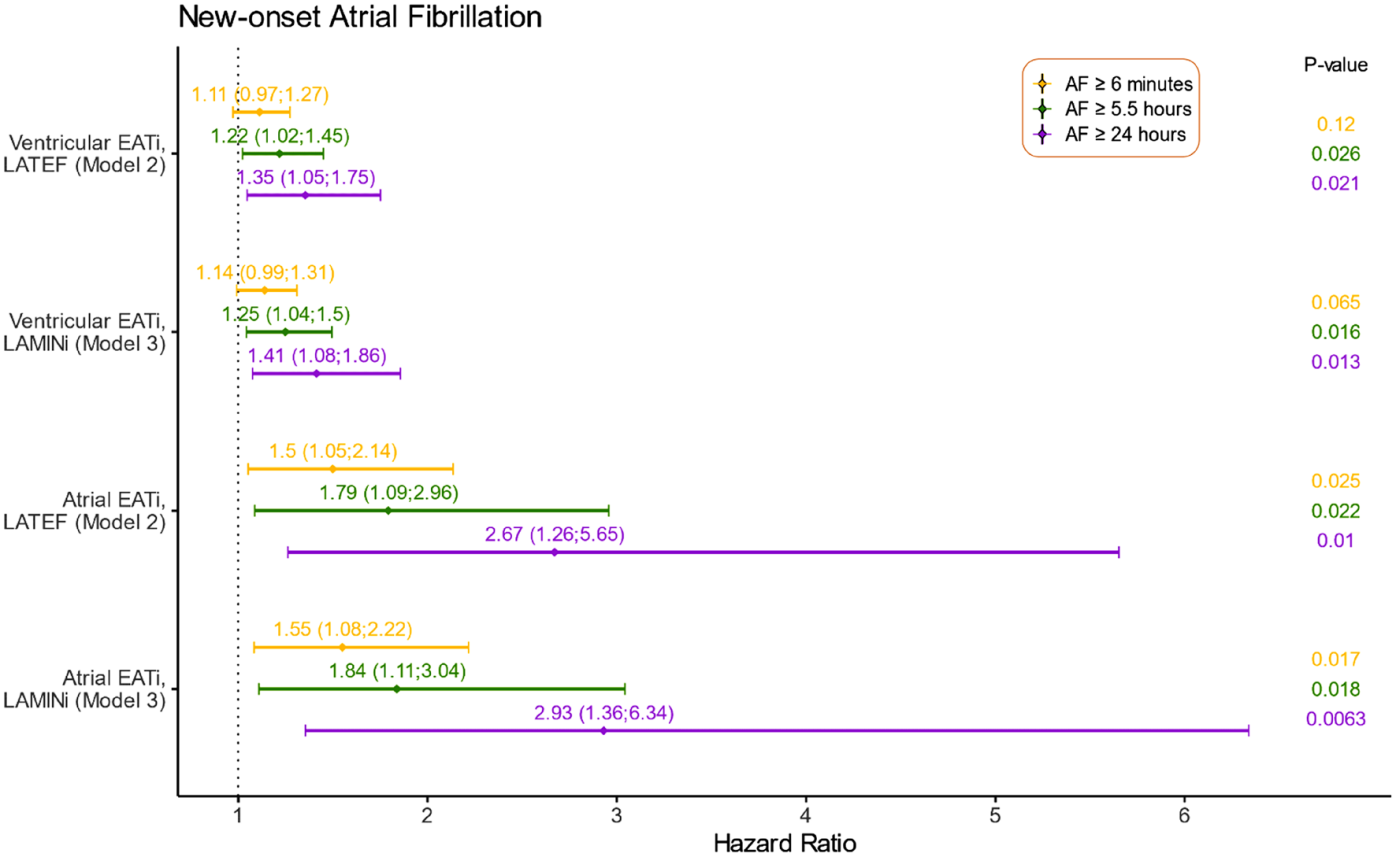
New-onset Atrial Fibrillation. This figure represents the hazard ratios (95% confidence intervals) for associations between epicardial adipose tissue (EAT) and the risk of developing new-onset AF episodes ≥ 6 min in yellow, ≥ 5.5 h in green, and ≥ 24 h in violet. The figure illustrates results from a cause-specific Cox model adjusted for age, sex, heart failure, diabetes, previous stroke, previous acute myocardial infarction, and/or coronary artery bypass graft surgery, cardiac valvular disease, and either left atrial total ejection fraction (LATEF), representing *Model 2*, or left atrial minimum volume (LAMIN), representing *Model 3*. Both EAT and LAMIN are indexed to body surface area (BSA) indicated by *i*

In models 2 and 3, after further adjustments with the inclusion of LATEF and LAMIN in separate analyses, the significant impact of EAT was maintained, as illustrated in Fig. 2. In the analysis with LAMIN, we see an increased risk with HR of 2.93 [CI (1.36–6.34); p = 0.01] of AF episodes ≥ 24 h with atrial EAT as an independent predictor. This increased risk was also seen for atrial EAT with regards to episodes of both ≥ 6 min, ≥ 5.5 h with HR of 1.55 [CI (1.08–2.22); p = 0.02] and 1.84 [CI (1.11–3.04); p = 0.02], respectively. Ventricular EAT elicits the same signals, though with lower HR: AF episodes ≥ 5.5 h and ≥ 24 h showed HR of 1.25 [CI (1.04–1.5); p = 0.02] and 1.41 [CI (1.08–1.86); p = 0.01], respectively (Table S6).

The same durations were significantly associated with EAT when adjusted for LATEF instead of LAMIN (Table S7). The strongest associations were seen ≥ 24 h for atrial EAT with HR 2.67 [CI (1.26–5.65); p = 0.01]] and ≥ 24 h for ventricular EAT with the HR 1.35 [CI (1.05–1.75); p = 0.02].

All models can be found in Supplementary material*.*

A simple overview of EAT’s impact on the mean time-to-incident AF episodes can also be found in Supplementary material (Table S8).

Furthermore, we performed risk prediction analyses, which can be found in Supplementary material (Table S9). In this analysis both atrial- and ventricular EAT significantly changes the area under the curve when using CHARGE-AF as reference. If only LAMIN or LATEF is compared to the reference, no significant change is found.

### Association between EAT and AF burden

Atrial EAT was weakly correlated with AF burden. When the burden was measured as a percentage, atrial EAT had an R = 0.23 (p = 0.043). Ventricular EAT did not show a significant correlation with AF burden. It should be noted that the median AF burden was quite low, and it should therefore only be viewed as hypothesis generating.

The impact of EAT on the AF burden, when divided into low/high EAT (according to the median value), is summarized in Supplementary material (Table S8).

## Discussion

We performed CMR in 203 participants from the LOOP study subsequently followed by more than 3 years of continuous monitoring. AF was detected in 38% of the participants [14, 15] and we found an association between the amount of EAT at inclusion and incident AF, which was most apparent for the group of participants with longer AF episodes.

Two recent large scale randomized clinical trials have investigated the effect of oral anticoagulation treatment in patients with subclinical AF. The NOAH-AFNET 6 trial with 2536 study participants followed for 21 months which was terminated prematurely did not show any reduction in the composite endpoint of cardiovascular death, stroke, or systemic embolism [18]. The ARTESIA trial, which included 4012 participants followed for 42 months, showed a significant reduction in stroke and systemic embolism, but also for severe stroke among participants treated with apixaban compared with aspirin [19]. A meta-analysis of the two trials found that anticoagulation for subclinical AF was efficacious in reducing strokes [20], strengthening the argument that subclinical AF could potentially be a relevant target for screening. Our study investigated the association between EAT and subclinical AF but due to its observational design it does not provide information regarding the treatment of subclinical AF. Instead, we have investigated the correlation from a theoretical perspective, and provide new insights to better understand the overall impact of EAT and correlations should be interpreted with caution.

To the best of our knowledge, the association between EAT and subclinical AF has not previously been investigated, though the relationship between EAT and clinical AF has been established. Previous studies have found EAT to be significantly associated with both symptomatic AF development, AF chronicity, and AF burden [11, 21–24]. Several studies have also found EAT volume and thickness to be more closely related to clinical AF than other obesity related parameters such as BMI, waist circumference and waist-to-hip ratio[6].

As seen in Fig. 2, both atrial- and ventricular EAT have a significant association with AF when adjusting for both left atrial size as determined by LAMIN and function as determined by LATEF. In previous studies these atrial remodeling markers have been shown to be significantly correlated to AF [16, 25]. When we adjust for these variables and various other clinical factors (CHA_2_DS_2_-VASc-score), EAT remains significantly associated with especially longer durations of subclinical AF. This supports the previous findings regarding EAT being an independent marker of AF, where the Framingham Heart Study was the first to report this significant association between pericardial adipose tissue and AF, also after correction for established risk factors [23]. Previous studies have adjusted for BMI, age, and other known clinical risk factors such as hypertension or congestive heart failure [23, 24]. To our knowledge, no previous study has adjusted for the atrial remodeling markers LAMIN and LATEF, and we hereby highlight the significance of EAT when correcting for even more known risk factors for AF. As described, there are multiple risk factors for AF, and this study does not intend to diminish the importance of these. In the LOOP study, pulse rate, blood pressure, and clinical comorbidities have all been associated with subclinical AF incidence, but we implement EAT as a marker, and have investigated how this measurement further describes the diverse and complex nature of subclinical AF.

Overall, the hazard ratios for increased EAT increase with longer durations of AF, as illustrated in Fig. 2 where atrial EAT seems to be superior compared to ventricular EAT. This tendency is also seen in the univariable analyses, though it did not show statistical significance. The univariable regression analyses do not reflect the diverse nature of AF. The analyses are too simple with a substantial number of omitted variables, which are well known risk factors, and we accordingly do not find it surprising that the univariable analyses did not demonstrate significance. EAT cannot solely explain AF without taking other risk factors into consideration, but as seen in the multivariable regressions, which constitutes a more reliable and thorough analysis, EAT adds value in the prediction of subclinical AF. It should be noted that the number of events decreases in the group with longer durations of AF episodes, and a larger scale study would be necessary to strengthen the association of this result. There seems to be a correlation between the severity of AF, in terms of episode durations, and how strong the predictions are. Several studies have previously found strong evidence of the importance of body weight in regard to the severity and recurrence of symptomatic AF [12, 26]. The association between AF and obesity becomes stronger when EAT is considered, and since longer durations of AF are more often seen in patients with obesity [12], it supports our findings, that EAT is more strongly correlated to longer AF durations, also regarding subclinical AF. This finding supports results from a CMR study from 2011, demonstrating a dose–response association between pericardial adipose tissue and AF severity (in regards to AF chronicity and AF burden) [21].

The association between EAT and AF burden has been investigated already in 2010 where cardiac computed tomography angiograms were used to measure the thickness of atrial EAT [24]. In this study, the authors found a significantly thicker layer of EAT in patients with persistent AF compared to paroxysmal AF or patients without AF.

In our study, we did not find any clear signal regarding EAT and AF burden when measured as a percentage. It should be noted that the median AF burden is very low, and the correlations to AF burden largely should be viewed as hypothesis generating, and a larger cohort would be necessary to strengthen the results. The duration of AF episodes could also be considered a measure of AF burden, and we hereby hypothesize that the same tendencies would be present, when AF burden as a percentage is investigated. Since we had a very low median AF burden further studies are necessary to fully understand the impact of EAT on the burden of subclinical AF.

The thickness of atrial EAT has been found to be more strongly correlated to AF compared to the thickness of ventricular EAT measured by computed tomography [27–30]. We have performed volumetric measurements on CMR images, which is an imaging method that has previously been validated in the assessment of pericardial adipose tissue [31, 32]. In this study, we also see tendencies supporting the superiority of atrial EAT regarding subclinical AF episodes, when compared to ventricular EAT, and we hereby substantiate the current knowledge and furthermore provide new knowledge about subclinical AF.

### Limitations

First, this study has a limited cohort size which decreases the power of the results, especially from AF episodes ≥ 24 h with a modest number of events of 15.

Also, as in any clinical situation, we cannot with certainty exclude the possibility that some participants suffered from asymptomatic paroxysmal AF at inclusion. Due to the increasing incidence of AF throughout the follow-up period in the original LOOP study, it does suggest that the majority of the AF episodes were truly new-onset. A strength of this study is that continuous monitoring for more than 3 years offers the possibility of detecting virtually all AF episodes.

It should be noted that these results apply to an at-risk population with participants of at least 70 years old at inclusion, and therefore cannot be applied directly to other populations.

## Conclusion

Atrial- and ventricular EAT assessed by CMR are significantly associated with incident subclinical AF. In this study, we found EAT to be an independent predictor of AF episodes ≥ 6 min, ≥ 5.5 h, and ≥ 24 h. EAT was an even stronger risk factor when considering the group of participants with longer AF episodes only.

### Supplementary Information

Below is the link to the electronic supplementary material.

Supplementary file1 (DOCX 62 KB)

Supplementary file2 (MOV 1159 KB)
