# Supplementary material for: Epicardial adipose tissue and subclinical incident atrial fibrillation as detected by continuous monitoring: a cardiac magnetic resonance imaging study
Source: Int J Cardiovasc Imaging. 2024 Jan 21;40(3):591–9. doi: 10.1007/s10554-023-03029-z (PMC10951027; doi:10.1007/s10554-023-03029-z)
Supplement: Supplementary file 1 — Supplementary file1 (DOCX 62 KB) [file 10554_2023_3029_MOESM1_ESM.docx]

# **Supplementary Material**

# **Data acquisition**

Steady-state free precession cine sequences [8mm; no gap; 25 phases; field of view (300–360) $x$ 360mm adjusted for each patient; matrix size (174–224) $x$ (138–224)], 10–15 s at end-expiratory breath-holds.

# **Tables**

Abbreviations used in the tables below:

- EAT: Epicardial adipose tissue
- BSA: Body surface area
- CHF: Congestive heart failure
- AMI: Acute myocardial infarction
- CABG: Coronary artery bypass surgery
- LAMIN: Left atrial minimum volume
- LAMAX: Left atrial maximum volume
- LATEF: Left atrial total ejection fraction
- min: Minutes
- h: Hours
- ml: Milliliter
- mm: Millimeter
- AUC: Area under the receiver operating characteristics curve
- ΔAUC: Difference in area under the receiver operating characteristics curve
- CHARGE-AF: Established AF prediction score for clinical AF

| Table s1. Interobserver and Intraobserver Variability | | | | | |
| --- | --- | --- | --- | --- | --- |
| LB | | **EG (1)** | | **EG (2)** | |
| Ventricular EAT (ml) | **Atrial EAT (ml)** | **Ventricular EAT (ml)** | **Atrial EAT (ml)** | **Ventricular EAT (ml)** | **Atrial EAT (ml)** |
| 181 | 94 | 182.3 | 81.7 | 188.5 | 69.4 |
| 114 | 54 | 114.2 | 45 | 126.4 | 46.2 |
| 100 | 69 | 104.6 | 56.7 | 99 | 60.6 |
| 126 | 50 | 111.9 | 36.9 | 110.2 | 46 |
| 52 | 15 | 53.2 | 16 | 45.3 | 13.2 |
| 107 | 27 | 96.5 | 21.9 | 102.9 | 30.2 |
| 60 | 36 | 75.7 | 23.9 | 67.7 | 15.8 |
| 116 | 26 | 128.1 | 25.3 | 129 | 26.4 |
| 84 | 29 | 83 | 26.6 | 87.2 | 30.8 |
| 59 | 27 | 57.4 | 21.4 | 65.7 | 30.4 |
| 102 | 39 | 118.6 | 41.3 | 108 | 45.8 |
| 97 | 23 | 79 | 19 | 79.3 | 21.8 |
| 89 | 29 | 97.7 | 26.3 | 94.2 | 30.5 |
| 63 | 33 | 71.1 | 22.5 | 94.6 | 42 |
| 87 | 33 | 79.4 | 27.1 | 80.1 | 32.1 |
| 83 | 42 | 89.3 | 32.6 | 92.2 | 36.6 |
| 71 | 21 | 60.4 | 26.6 | 59.7 | 23.9 |
| 76 | 27 | 99.9 | 29.2 | 101.1 | 26.7 |
| 104 | 46 | 91.2 | 37.7 | 98.6 | 33.3 |
| 75 | 29 | 82.4 | 41.5 | 91 | 39.2 |

The authors Litten Bertelsen (LB) and Eva Guldberg (EG) have performed the following measurements to investigate the interobserver and intraobserver agreement.

EAT is indexed to BSA as indicated by *i.*

| **Table s2. Baseline Characteristics – atrial EAT** | | | | |
| --- | --- | --- | --- | --- |
| **Variable** | **Small amount of atrial EATi** | **Large amount of atrial EATi** | **Total** | **P-value** |
| Male, n (%) | 64 (62.7) | 64 (63.4) | 129 (63.2) | 0.74 |
| Age (years), mean (SD) | 78 (4.4) | 74.4 (3.3) | 76.2 (4.2) | <0.01 |
| **Medical history** |  |  |  |  |
| Hypertension, n (%) | 88 (86.3) | 96 (95.0) | 185 (90.7) | 0.09 |
| Diabetes mellitus, n (%) | 27 (26.5) | 36 (35.6) | 64 (31.4) | 0.12 |
| Congestive heart failure, n (%) | 4 (3.9) | 3 (3.0) | 7 (3.4) | 0.92 |
| Previous stroke, n (%) | 22 (21.6) | 17 (16.8) | 39 (19.1) | 0.61 |
| Previous transient ischemic attack, n (%) | 14 (13.7) | 9 (8.9) | 23 (11.3) | 0.52 |
| Previous acute myocardial infarction, n (%) | 7 (6.9) | 12 (11.9) | 19 (9.3) | 0.45 |
| Previous CABG, n (%) | 4 (3.9) | 5 (5.0) | 9 (4.4) | 0.92 |
| Valvular disease, n (%) | 6 (5.9) | 1 (1.0) | 8 (3.9) | <0.01 |
| COPD, n (%) | 5 (4.9) | 7 (6.9) | 12 (5.9) | 0.80 |
| CHA2DS2-VASc-score, mean (SD) | 3 (1.3) | 2.7 (1.1) | 2.9 (1.2) | 0.04 |
| **Medical treatment** |  |  |  |  |
| Beta blockers n (%) | 11 (10.8) | 29 (28.7) | 41 (20.1) | <0.01 |
| Calcium blockers n (%) | 41 (40.2) | 37 (36.6) | 78 (38.2) | 0.64 |
| Renin-angiotensin-system medication n (%) | 51 (50.0) | 68 (67.3) | 120 (58.8) | 0.03 |
| Statins, n (%) | 50 (49.0) | 63 (62.4) | 114 (55.9) | 0.11 |
| Diuretics, n (%) | 36 (35.3) | 29 (28.7) | 65 (31.9) | 0.48 |
| Platelet inhibitors, n (%) | 56 (54.9) | 51 (50.5) | 107 (52.5) | 0.47 |
| Antidiabetics, n (%) | 24 (23.5) | 32 (31.7) | 57 (27.9) | 0.12 |
| **Physical examination** |  |  |  |  |
| Systolic blood pressure (mmHg), mean (SD) | 151.4 (16.8) | 145 (18.2) | 148.3 (17.8) | <0.01 |
| Diastolic blood pressure (mmHg), mean (SD) | 84.1 (10.5) | 84.2 (11.5) | 84.1 (10.9) | 0.93 |
| Pulse rate (bpm), mean (SD) | 70.3 (11.1) | 71.7 (12.7) | 70.9 (11.9) | 0.41 |
| Height (cm), mean (SD) | 171.9 (9.3) | 172.3 (8.7) | 172.1 (9) | 0.76 |
| Weight (kg), mean (SD) | 79.4 (13.8) | 87.6 (16.3) | 83.5 (15.6) | <0.01 |
| BMI (kg/cm2), mean (SD) | 26.8 (4.1) | 29.5 (5) | 28.1 (4.7) | <0.01 |

EAT is indexed to BSA as indicated by *i.*

| **Table s3. Baseline Characteristics – ventricular EAT** | | | | |
| --- | --- | --- | --- | --- |
| **Variable** | **Small amount of ventricular EATi** | **Large amount of ventricular EATi** | **Total** | **P-value** |
| Male, n (%) | 60 (58.8) | 68 (67.3) | 129 (63.2) | 0.34 |
| Age (years), mean (SD) | 77.1 (4.5) | 75.2 (3.7) | 76.2 (4.2) | <0.01 |
| **Medical history** |  |  |  |  |
| Hypertension, n (%) | 89 (87.3) | 95 (94.1) | 185 (90.7) | 0.24 |
| Diabetes mellitus, n (%) | 27 (26.5) | 36 (35.6) | 64 (31.4) | 0.12 |
| Congestive heart failure, n (%) | 1 (1.0) | 6 (5.9) | 7 (3.4) | 0.15 |
| Previous stroke, n (%) | 23 (22.5) | 16 (15.8) | 39 (19.1) | 0.42 |
| Previous transient ischemic attack, n (%) | 14 (13.7) | 9 (8.9) | 23 (11.3) | 0.52 |
| Previous acute myocardial infarction, n (%) | 9 (8.8) | 10 (9.9) | 19 (9.3) | 0.92 |
| Previous CABG, n (%) | 4 (3.9) | 5 (5.0) | 9 (4.4) | 0.92 |
| Valvular disease, n (%) | 3 (2.9) | 4 (4.0) | 8 (3.9) | <0.01 |
| COPD, n (%) | 4 (3.9) | 8 (7.9) | 12 (5.9) | 0.47 |
| CHA2DS2-VASc-score, mean (SD) | 3 (1.3) | 2.8 (1.2) | 2.9 (1.2) | 0.26 |
| **Medical treatment** |  |  |  |  |
| Beta blockers n (%) | 14 (13.7) | 26 (25.7) | 41 (20.1) | 0.01 |
| Calcium blockers n (%) | 38 (37.3) | 40 (39.6) | 78 (38.2) | 0.69 |
| Renin-angiotensin-system medication n (%) | 48 (47.1) | 71 (70.3) | 120 (58.8) | <0.01 |
| Statins, n (%) | 51 (50.0) | 62 (61.4) | 114 (55.9) | 0.18 |
| Diuretics, n (%) | 30 (29.4) | 35 (34.7) | 65 (31.9) | 0.57 |
| Platelet inhibitors, n (%) | 54 (52.9) | 53 (52.5) | 107 (52.5) | 0.57 |
| Antidiabetics, n (%) | 24 (23.5) | 32 (31.7) | 57 (27.9) | 0.12 |
| **Physical examination** |  |  |  |  |
| Systolic blood pressure (mmHg), mean (SD) | 150.2 (18.7) | 146.1 (16.6) | 148.3 (17.8) | 0.10 |
| Diastolic blood pressure (mmHg), mean (SD) | 84.8 (11) | 83.5 (10.9) | 84.1 (10.9) | 0.43 |
| Pulse rate (bpm), mean (SD) | 70.5 (11.1) | 71.4 (12.7) | 70.9 (11.9) | 0.57 |
| Height (cm), mean (SD) | 171 (9.5) | 173.3 (8.4) | 172.1 (9) | 0.07 |
| Weight (kg), mean (SD) | 79.1 (13.5) | 87.8 (16.4) | 83.5 (15.6) | <0.01 |
| BMI (kg/cm2), mean (SD) | 27.1 (4.6) | 29.2 (4.7) | 28.1 (4.7) | <0.01 |

The following models illustrate the hazard ratio, confidence interval, and p-value.

All volumes indexed to BSA are indicated by *i.*

| Table s4. Univariable Cox Regression | | | | |
| --- | --- | --- | --- | --- |
|  | 6 min to 5.5 h | 5.5 h to 24 h | 24 h or longer |  |
| EATi per 10 ml* | 1.12 (0.9-1.5); 0.47 | 1.09 (0.73-1.64); 0.67 | 1.48 (0.81-2.69); 0.2 | **Atria** |
|  |  |  |  |  |
| EATi per 10 ml* | 1.05 (0.92-1.18); 0.49 | 1.06 (0.91-1.24); 0.44 | 1.18 (0.96-1.46); 0.12 | **Ventricle** |

| Table s5. Model 1: Multivariable Cox Regression - Baseline Characteristics | | | | |
| --- | --- | --- | --- | --- |
|  | 6 min to 5.5 h | 5.5 h to 24 h | 24 h or longer |  |
| Age in years per 5 years** | 1.23 (0.93-1.63); 0.14 | 1.41 (0.96-2.07); 0.08 | 1.68 (0.92-3.08); 0.09 | **Atria** |
| Sex | 0.85 (0.52-1.37); 0.5 | 0.66 (0.33-1.29); 0.22 | 0.92 (0.29-2.85); 0.88 |  |
| CHF | 0.75 (0.22-2.59); 0.65 | 0.81 (0.17-3.8); 0.78 | 0.98 (0.1-9.45); 0.99 |  |
| Hypertension | 0.85 (0.37-1.99); 0.71 | **0.35 (0.13-0.95); 0.04** | 0.46 (0.09-2.34); 0.35 |  |
| Diabetes | 0.96 (0.57-1.61); 0.86 | 0.96 (0.46-1.98); 0.91 | 0.76 (0.23-2.49); 0.65 |  |
| Stroke | 1.69 (0.98-2.92); 0.06 | **3.4 (1.62-7.1); <0.01** | 2.19 (0.62-7.74); 0.23 |  |
| AMI or CABG | 1.23 (0.58-2.59); 0.59 | 2.29 (0.88-5.91); 0.09 | 0.53 (0.07-4.21); 0.54 |  |
| Valvular disease | **2.9 (1.1-7.67); 0.03** | **5.24 (1.65-16.66); <0.01** | 2.93 (0.31-28); 0.35 |  |
| EATi per 10 ml | 1.34 (0.95-1.88); 0.1 | **1.63 (1.0-2.64); 0.05** | **2.23 (****1.1-4.51); 0.03** |  |
|  |  |  |  |  |
| Age in years per 5 years** | 1.17 (0.89-1.52); 0.26 | 1.37 (0.9-1.87); 0.17 | 1.46 (0.81-2.63); 0.21 | **Ventricle** |
| Sex | 0.85 (0.53-1.38); 0.52 | 0.64 (0.35-1.33); 0.26 | 1.01 (0.32-3.14); 0.99 |  |
| CHF | 0.77 (0.23-2.64); 0.68 | 0.7 (0.16-3.59); 0.73 | 0.91 (0.09-9.06); 0.94 |  |
| Hypertension | 0.89 (0.38-2.07); 0.78 | **0.35 (0.14-0.99); 0.05** | 0.49 (0.1-2.52); 0.4 |  |
| Diabetes | 0.95 (0.56-1.61); 0.86 | 0.95 (0.46-1.99); 0.91 | 0.71 (0.21-2.42); 0.59 |  |
| Stroke | 1.67 (0.97-2.87); 0.06 | **3.37 (1.62-7.12); <0.01** | 1.99 (0.57-6.97); 0.28 |  |
| AMI or CABG | 1.34 (0.64-2.81); 0.44 | **2.7 (1.04-7.01); 0.04** | 0.68 (0.09-5.43); 0.72 |  |
| Valvular disease | 2.55 (0.98-6.61); 0.05 | **4.58 (1.45-13.26); 0.01** | 2.07 (0.29-18.25); 0.51 |  |
| EATi per 10 ml* | 1.09 (0.95-1.24); 0.23 | **1.09** **(****1.0-1.41); 0.05** | **1.****29 (****1.0-1.65); 0.05** |  |
| * Hazard ratio increases per 10 ml increase of EAT. Every time the amount of EAT increases by 10 ml, the risk of getting new-onset AF in the specific time duration also increases.  ** Hazard ratio increases per 5-year increase in age. Every time the age increases by 5 years, the risk of getting new-onset AF in the specific time duration also increases. | | | | |

| Table s6. Model 2: Multivariable Cox Regression - Baseline Characteristics + LAMIN | | | | |
| --- | --- | --- | --- | --- |
|  | 6 min to 5.5 h | 5.5 h to 24 h | 24 h or longer |  |
| Age in years per 5 years** | 1.22 (0.93-1.61); 0.16 | 1.4 (0.96-2.06); 0.08 | 1.78 (0.97-3.27); 0.06 | **Atria** |
| Sex | 0.75 (0.47-1.22); 0.25 | 0.61 (0.31-1.19); 0.15 | 0.76 (0.24-2.45); 0.65 |  |
| CHF | 0.68 (0.19-2.39); 0.55 | 0.77 (0.16-3.69); 0.74 | 0.85 (0.08-8.72); 0.89 |  |
| Hypertension | 0.68 (0.29-1.61); 0.38 | **0.3 (0.11-0.83); 0.02** | 0.32 (0.06-1.75); 0.19 |  |
| Diabetes | 1.02 (0.6-1.72); 0.95 | 1.1 (0.52-2.3); 0.81 | 0.84 (0.24-2.88); 0.78 |  |
| Stroke | 1.66 (0.96-2.88); 0.07 | **3.36 (1.59-7.12); <0.01** | 1.82 (0.49-6.82); 0.37 |  |
| AMI or CABG | 1.27 (0.6-2.66); 0.53 | 2.13 (0.82-5.53); 0.12 | 0.45 (0.06-3.62); 0.46 |  |
| Valvular disease | **3.52 (1.31-9.44); 0.01** | **5.78 (1.79-18.59); <0.01** | 3.7 (0.35-38.76); 0.27 |  |
| EATi per 10 ml* | **1.55 (1.08-2.22); 0.02** | **1.84 (1.11-3.04); 0.02** | **2.93** **(1.36-6.34); 0.01** |  |
| LAMINi | **1.05 (1.02-1.07); <0.01** | **1.04 (1.01-1.07); 0.01** | **1.06 (1.01-1.11); 0.01** |  |
|  |  |  |  |  |
| Age in years per 5 years** | 1.13 (0.87-1.47); 0.36 | 1.27 (0.88-1.82); 0.21 | 1.45 (0.81-2.6); 0.21 | **Ventricle** |
| Sex | 0.76 (0.47-1.23); 0.26 | 0.63 (0.32-1.24); 0.18 | 0.84 (0.26-2.7); 0.78 |  |
| CHF | 0.72 (0.21-2.5); 0.61 | 0.73 (0.15-3.47); 0.69 | 0.76 (0.07-8.18); 0.82 |  |
| Hypertension | 0.71 (0.3-1.69); 0.44 | **0.32 (0.12-0.87); 0.03** | 0.35 (0.06-1.92); 0.23 |  |
| Diabetes | 0.99 (0.58-1.68); 0.96 | 1.07 (0.51-2.25); 0.87 | 0.72 (0.2-2.58); 0.61 |  |
| Stroke | 1.66 (0.96-2.87); 0.07 | **3.46 (1.62-7.4); <0.01** | 1.72 (0.46-6.4); 0.42 |  |
| AMI or CABG | 1.49 (0.71-3.11); 0.29 | **2.72 (1.05-7.05); 0.04** | 0.68 (0.08-5.4); 0.71 |  |
| Valvular disease | **2.87 (1.09-7.53); 0.03** | **4.67 (1.53-14.27); 0.01** | 2.4 (0.25-22.75); 0.45 |  |
| EATi per 10 ml* | 1.14 (0.99-1.31); 0.07 | **1.25 (1.04-1.5); 0.02** | **1.41 (1.08-1.86); 0.01** |  |
| LAMINi | **1.05 (1.02-1.07); <0.01** | **1.04 (1.01-1.07); 0.01** | **1.06 (1.01-1.12); 0.01** |  |
| * Hazard ratio increases per 10 ml increase of EAT. Every time the amount of EAT increases by 10 ml, the risk of getting new-onset AF in the specific time duration also increases.  ** Hazard ratio increases per 5-year increase in age. Every time the age increases by 5 years, the risk of getting new-onset AF in the specific time duration also increases. | | | | |

| Table s7. Model 3: Multivariable Cox Regression - Baseline Characteristics + LATEF | | | | |
| --- | --- | --- | --- | --- |
|  | 6 min to 5.5 h | 5.5 h to 24 h | 24 h or longer |  |
| Age in years per 5 years** | 1.1 (0.91-1.47); 0.5 | 1.29 (0.87-1.92); 0.21 | 1.57 (0.84-2.91); 0.16 | **Atria** |
| Sex | 0.85 (0.53-1.37); 0.5 | 0.66 (0.34-1.3); 0.23 | 0.9 (0.28-2.85); 0.86 |  |
| CHF | 0.51 (0.14-1.78); 0.29 | 0.61 (0.13-2.93); 0.54 | 0.56 (0.05-5.92); 0.63 |  |
| Hypertension | 0.57 (0.24-1.36); 0.21 | **0.26 (0.09-0.72); 0.01** | 0.3 (0.05-1.68); 0.17 |  |
| Diabetes | 0.88 (0.52-1.5); 0.65 | 0.96 (0.46-2.01); 0.91 | 0.7 (0.20-2.4); 0.57 |  |
| Stroke | 1.53 (0.88-2.66); 0.13 | **3.15 (1.47-6.76); <0.01** | 1.7 (0.44-6.48); 0.44 |  |
| AMI or CABG | 1.39 (0.66-2.94); 0.38 | 2.36 (0.91-6.11); 0.08 | 0.55 (0.07-4.29); 0.57 |  |
| Valvular disease | **4.1 (1.52-11.05); <0.01** | **6.53 (2.01-21.17); <0.01** | 4.22 (0.42-42.72); 0.22 |  |
| EATi per 10 ml* | **1.5 (1.05-2.14); 0.02** | **1.79 (1.09-2.96); 0.02** | **2.67** **(1.26-5.65); 0.01** |  |
| LATEF | **0.92 (0.89-0.95); <0.01** | **0.93 (0.89-0.98); <0.01** | **0.91 (0.85-0.99); 0.02** |  |
|  |  |  |  |  |
| Age in years per 5 years** | 1.02 (0.78-1.34); 0.88 | 1.17 (0.8-1.71); 0.43 | 1.29 (0.71-2.35); 0.4 | **Ventricle** |
| Sex | 0.85 (0.53-1.37); 0.51 | 0.7 (0.36-1.37); 0.29 | 1.01 (0.32-3.24); 0.98 |  |
| CHF | 0.54 (0.16-1.9); 0.34 | 0.59 (0.12-2.81); 0.51 | 0.54 (0.05-5.72); 0.61 |  |
| Hypertension | 0.6 (0.25-1.44); 0.26 | **0.27 (0.1-0.76); 0.01** | 0.32 (0.06-1.79); 0.2 |  |
| Diabetes | 0.87 (0.51-1.49); 0.61 | 0.93 (0.44-1.97); 0.85 | 0.62 (0.17-2.27); 0.47 |  |
| Stroke | 1.52 (0.87-2.64); 0.14 | **3.18 (1.48-6.85); <0.01** | 1.55 (0.41-5.84); 0.52 |  |
| AMI or CABG | 1.59 (0.76-3.34); 0.22 | **2.89 (1.11-7.53); 0.03** | 0.74 (0.09-5.89); 0.78 |  |
| Valvular disease | **3.38 (1.28-8.95); 0.01** | **5.26 (1.71-16.19); <0.01** | 2.77 (0.3-25.59); 0.37 |  |
| EATi per 10 ml* | 1.11 (0.97-1.27); 0.12 | **1.22 (1.02-1.45); 0.03** | **1.35 (1.05-1.75); 0.02** |  |
| LATEF | **0.92 (0.89-0.95); <0.01** | **0.93 (0.89-0.98); <0.01** | **0.91 (0.85-0.99); 0.03** |  |
| * Hazard ratio increases per 10 ml increase of EAT. Every time the amount of EAT increases by 10 ml, the risk of getting new-onset AF in the specific time duration also increases.  ** Hazard ratio increases per 5-year increase in age. Every time the age increases by 5 years, the risk of getting new-onset AF in the specific time duration also increases. | | | | |

| Table s8. Overview of AF Burden and AF Episodes | | | | | | | | |
| --- | --- | --- | --- | --- | --- | --- | --- | --- |
|  | **Atrial EAT** | | | | **Ventricular EAT** | | | |
|  | **Low atrial EATi***** | **High atrial EATi** | **Total** | **p-value** | **Low ventricular EATi****** | **High ventricular EATi** | **Total** | **p-value** |
| n | 102 | 101 | 203 |  | 102 | 101 | 203 |  |
| Incidents of AF (%) | 67 (65.7) | 57 (56.4) | 124 (61.1) | 0.23 | 60 (58.8) | 64 (63.4) | 124 (61.1) | 0.6 |
| AF burden (%), median [IQR] | 0.1 [0.0, 0.2] | 0.2 [0.1, 1.8] | 0.1 [0.1, 1.0] | 0.1 | 0.1 [0.0, 0.2] | 0.4 [0.1, 3.6] | 0.1 [0.1, 1.0] | <0.01 |
| Number of incidents of AF lasting at least 6 min | 34 | 44 | 78 |  | 41 | 37 | 78 |  |
| Days until AF lasting at least 6 min, mean (SD) | 930.6 (455.8) | 799.4 (520.5) | 865 (492.5) | 0.06 | 847.7 (472.7) | 882.3 (513.2) | 865 (492.5) | 0.62 |
| Number of incidents of AF lasting at least 5.5h | 15 | 25 | 40 |  | 15 | 25 | 40 |  |
| Days until AF lasting at least 5.5h, mean (SD) | 1123.6 (283.4) | 1012.3 (408.1) | 1068 (354.9) | 0.02 | 1094.7 (291.3) | 1041.3 (408.5) | 1068 (354.9) | 0.28 |
| Number of incidents of AF lasting at least 24h | 4 | 11 | 15 |  | 2 | 13 | 15 |  |
| Days until AF lasting at least 24h, mean (SD) | 1170.4 (224.3) | 1121.8 (298.4) | 1146.1 (264.4) | 0.19 | 1155.5 (221.6) | 1136.7 (302) | 1146.1 (264.4) | 0.61 |
| *** Atrial EAT is divided into low/high according to the median value.  **** Ventricular EAT is divided into low/high according to the median value. | | | | | | | | |

| Table s9. Risk Prediction Analyses | | | | |
| --- | --- | --- | --- | --- |
|  | **Model** | **AUC (CI)** | **ΔAUC (CI)** | **p-value** |
| AF ≥ 6 minutes | CHARGE_AF (reference) | 0.56 (0.457-0.662) |  |  |
|  | CHARGE_AF + LAMINi | 0.655 (0.555-0.755) | 0.095 (-0.018-0.209) | 0.1 |
|  | CHARGE_AF + LATEF | 0.681 (0.583-0.778) | 0.121 (-0.04-0.246) | 0.057 |
|  | **CHARGE_AF + LAMINi + Ventricular EATi** | **0.668 (0.57-0.767)** | **0.109 (0.005-0.213)** | **0.04** |
|  | **CHARGE_AF + LAMINi + Atrial EATi** | **0.666 (0.567-0.766)** | **0.107 (0.001-0.213)** | **0.048** |
|  | **CHARGE_AF + LATEF**  **+ Ventricular EATi** | **0.69 (0.593-0.786)** | **0.13 (0.011-0.249)** | **0.032** |
|  | **CHARGE_AF + LATEF**  **+ Atrial EATi** | **0.684 (0.586-0.781)** | **0.124 (0.006-0.243)** | **0.04** |
| AF ≥ 5.5 hours | CHARGE_AF (reference) | 0.558 (0.438-0.678) |  |  |
|  | CHARGE_AF + LAMINi | 0.638 (0.527-0.75) | 0.08 (-0.194-0.033) | 0.165 |
|  | CHARGE_AF + LATEF | 0.666 (0.553-0.778) | 0.108 (-0.23-0.014) | 0.084 |
|  | CHARGE_AF + LAMINi + Ventricular EATi | 0.652 (0.54-0.764) | 0.094 (-0.204-0.015) | 0.091 |
|  | CHARGE_AF + LAMINi + Atrial EATi | 0.645 (0.535-0.756) | 0.087 (-0.195-0.02) | 0.112 |
|  | **CHARGE_AF + LATEF**  **+ Ventricular EATi** | **0.675 (0.565-0.786)** | **0.118 (-0.235-0.001)** | **0.05** |
|  | **CHARGE_AF + LATEF**  **+ Atrial EATi** | **0.676 (0.566-0.786)** | **0.118 (-0.235- -0.002)** | **0.046** |
| AF ≥ 24 hours | CHARGE_AF (reference) | 0.611 (0.428-0.794) |  |  |
|  | CHARGE_AF + LAMINi | 0.71 (0.565-0.855) | 0.099 (-0.259-0.061) | 0.225 |
|  | CHARGE_AF + LATEF | 0.718 (0.563-0.872) | 0.107 (-0.262-0.048) | 0.177 |
|  | **CHARGE_AF + LAMINi + Ventricular EATi** | **0.795 (0.679-0.91)** | **0.184 (-0.34- -0.028)** | **0.021** |
|  | **CHARGE_AF + LAMINi + Atrial EATi** | **0.766 (0.647-0.884)** | **0.155 (-0.305- -0.005)** | **0.043** |
|  | **CHARGE_AF + LATEF**  **+ Ventricular EATi** | **0.756 (0.621-0.89)** | **0.145 (-0.287- -0.003)** | **0.046** |
|  | **CHARGE_AF + LATEF**  **+ Atrial EATi** | **0.765 (0.634-0.895)** | **0.154 (-0.295- -0.012)** | **0.033** |
| We used cause-specific Cox regression models to analyze the risk of getting AF episodes lasting ≥ 6 minutes, ≥ 5.5 hours, and ≥ 24 hours. The area under the receiver operating characteristics curve (AUC) was calculated with the reference based on CHARGE AF. | | | | |
